# Fluoroquinolone Resistance in *Salmonella enterica* Serotype Choleraesuis, Taiwan, 2000–2003

**DOI:** 10.3201/eid1009.030596

**Published:** 2004-09

**Authors:** Cheng-Hsun Chiu, Tsu-Lan Wu, Lin-Hui Su, Jien-Wei Liu, Chishih Chu

**Affiliations:** * Chang Gung Children's Hospital, Taoyuan, Taiwan;; † Chang Gung Memorial Hospital, Taoyuan, Taiwan;; ‡ Chang Gung Memorial Hospital, Kaohsiung, Taiwan

**Keywords:** dispatch, Salmonella enterica serotype Choleraesuis, fluoroquinolone resistance, Taiwan

## Abstract

*Salmonella enterica* serotype Choleraesuis is a highly invasive pathogen that infects humans and causes systemic infections that require antimicrobial therapy. Surveillance in Taiwan showed that fluoroquinolone resistance in *S*. Choleraesuis markedly increased from 2000 to 2003, reaching approximately 70% in 2003.

Of the more than 2,000 nontyphoid *Salmonella* serotypes, *Salmonella enterica* serotype Choleraesuis is extremely invasive and usually associated with bacteremia in humans. Before 1999, this serotype was susceptible to fluoroquinolones (*1*). Since 2000, we have recorded rapidly increasing resistance to ciprofloxacin in *S*. Choleraesuis isolated from both human and swine sources in Taiwan (*2*). This finding is a cause for concern because fluoroquinolones are first-line drugs to treat systemic, nontyphoid salmonellosis. We conducted a prospective, laboratory-based surveillance study of fluoroquinolone resistance in *S*. Choleraesuis isolated from humans in four major teaching hospitals across Taiwan from 2000 to 2003.

## The Study

**Figure 1:**
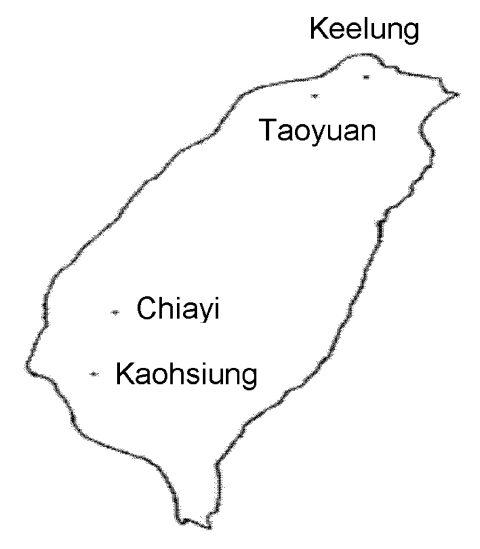
Geographic position of the four hospitals in Taiwan where *Salmonella enterica* Choleraesuis isolates were collected and surveyed for their susceptibility to ciprofloxacin, 2000–2003.

Chang Gung Memorial Hospital in Keelung is a 1,000-bed hospital. Chang Gung Memorial Hospital in Taoyuan, which includes Chang Gung Children's Hospital, is located in Taoyuan and has a capacity of 4,000 (which includes an additional 500 beds for Chang Gung Children's Hospital). Chang Gung Memorial Hospital in Kaohsiung, in southern metropolitan Taiwan, is a 2,000-bed hospital. Chang Gung Memorial Hospital in Chiayi, which was open for service in December 2001, is an 800-bed hospital. All four are teaching hospitals affiliated with Chang Gung University in Taoyuan, Taiwan. The geographic locations of these hospitals are shown in Figure 1. All *S*. Choleraesuis isolates were cultured and identified according to standard methods (*3*) in the clinical microbiologic laboratories of the four hospitals. No major changes were made in the policy concerning identification of *Salmonella* during the study years. *Salmonella* isolates were first checked with O antisera (Becton Dickinson and Co., Franklin Lakes, NJ) for their serogroups by the slide agglutination method. Because it causes invasive infections, *S*. Choleraesuis was specifically identified if a negative result was found in the citrate utilization test for any isolate identified as serogroup C1 *Salmonella*. H antiserum has been used to verify the accuracy of this procedure in our previous studies (*1**,**2*). The susceptibility to ciprofloxacin of the isolates was investigated by a standard disk-diffusion method (*4*). Susceptible or resistant isolates were defined according to the criteria suggested by the National Committee for Clinical Laboratory Standards (*4*); isolates in the intermediate category were deemed as resistant in this study.

**Figure 2:**
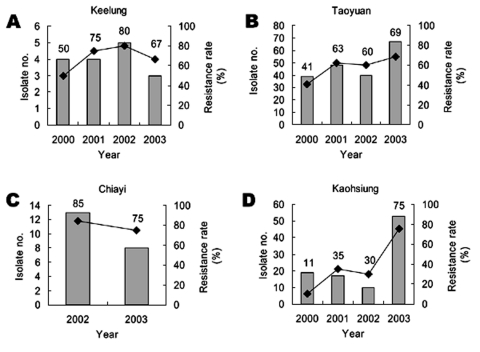
The annual isolate number (in bars) and the rate of ciprofloxacin resistance (in lines) among the isolates from the four hospitals in which *Salmonella enterica* Choleraesuis isolates were collected and surveyed for their susceptibility to ciprofloxacin, 2000–2003.

The annual isolate number of *S*. Choleraesuis among the four Chang Gung Memorial Hospitals and the resistance to ciprofloxacin from 2000 to 2003 are summarized in Figure 2. An apparent growing trend of resistance was observed. The overall resistance rate of the isolates collected from the four hospitals increased from 32.3% in 2000 to 56.5% in 2001, 61.8% in 2002, and 71.8% in 2003 (p < 0.00001 by chi-square test). Ciprofloxacin resistance rate was generally lower in 2000; however, such resistance increased to >50% in Keelung, Taoyuan, and the newly opened Chiayi Chang Gung Memorial Hospitals in 2001 to 2002. The resistance rate remained constantly high in 2003. The highest rate was found in clinical isolates from Chang Gung Memorial Hospital in Chiayi: 11 (85%) of 13 isolates were resistant in 2002. Although approximately 30% of the *S*. Choleraesuis isolates were resistant to ciprofloxacin in Chang Gung Memorial Hospital, Kaohsiung, before 2002, an upsurge to 76% was detected in this hospital in 2003.

## Conclusions

*S*. Choleraesuis is an infrequent serotype of *Salmonella* and has been isolated from humans in the United States (*5*). The annual reported number of *S*. Choleraesuis isolates from humans was approximately 80 from 1990 to 1996. The number decreased gradually thereafter, with an annual isolate number of 49 in 1997, 36 in 1998, 34 in 1999, and 15 in 2000 (*5*).

However, this highly invasive serotype is of particular concern in Taiwan; it was the second most common *Salmonella* isolate found in humans in two independent epidemiologic surveys (*6**,**7*). The reason for the difference between Taiwan and United States is an issue of interest. *S*. Choleraesuis is highly host-adapted to pigs. We have found that *S*. Choleraesuis isolates from humans and swine had the same or similar DNA fingerprints, which suggests that human infections were derived from pigs (*2*). Such infection likely arose as a result of the contamination of a food or water source. Eating pig offal by the local population likely contributed to the high prevalence of *S*. Choleraesuis infection in some areas of Taiwan.

Results of this surveillance study reflect the current status of fluoroquinolone resistance in *S*. Choleraesuis in Taiwan. The four hospitals surveyed in this study are located in different regions across Taiwan, the surveillance is prospective and longitudinal in design, and the overall isolate number examined is large enough to draw a conclusion. Minor geographic variation in the rate of ciprofloxacin resistance was observed. Among the four hospitals surveyed, Chang Gung Memorial Hospital in Kaohsiung is the only one located in a metropolitan area. Sanitation in a metropolitan area is better than that in the rural or country areas. The spread of resistant clones, therefore, may be slower in this area, as reflected by the lower number of *S*. Choleraesuis infections treated in Kaohsiung Chang Gung Memorial Hospital compared to those treated in Linko Chang Gung Memorial Hospital. Moreover, most pig farms are not located in large cities. These factors might explain why the spread of resistance in Kaohsiung was slower than in other regions before 2002. In contrast, isolates from Chiayi Chang Gung Memorial Hospital, which is located in the underdeveloped, southwestern coastal region of Taiwan, showed the highest rate of resistance to ciprofloxacin in 2002. The up-to-date data obtained in 2003 confirmed a wide dissemination of ciprofloxacin-resistant *S*. Choleraesuis across Taiwan.

We have found that all of the resistant strains carried mutations that give rise to the substitution of phenylalanine for serine at position 83 and asparagine for aspartic acid at position 87 in GyrA (*2*). In addition, mutations leading to an amino acid change from serine to isoleucine at position 80 in ParC were demonstrated in the ciprofloxacin-resistant *S*. Choleraesuis isolates (*8**,**9*). The emergence of fluoroquinolone resistance in *S*. Choleraesuis was mainly due to the dissemination of an endemic, resistant clone (*2**,**9*).

The emergence of fluoroquinolone resistance in this highly invasive nontyphoid *Salmonella* serotype represents a serious threat to public health and clinical medicine. Most of the ciprofloxacin-resistant *S*. Choleraesuis were also multidrug resistant to the conventional antimicrobial agents (*1**,**2**,**6**,**10*). This resistance indicates that extended-spectrum cephalosporins become the only reliable agent in the treatment of systemic infections caused by *S*. Choleraesuis. However, in 2002, we isolated for the first time from a patient with sepsis, a strain of *S*. Choleraesuis that was simultaneously resistant to ceftriaxone and ciprofloxacin (*8*). The ceftriaxone resistance gene, *bla*_CMY-2_, of this isolate was found on a potentially transmissible plasmid (*8*); thus, the spread of such resistance phenotype may be unavoidable in the future. In view of the severe situation, restricted use of antimicrobial agents in both humans and domestic animals as well as actively monitoring *S*. Choleraesuis isolates for antimicrobial drug resistance should be reinforced.

## References

[1] Su LH, Chiu CH, Kuo AJ, Chia JH, Sun CF, Leu HS, Secular trends in incidence and antimicrobial resistance among clinical isolates of *Salmonella* at a university hospital in Taiwan, 1983–1999. Epidemiol Infect. 2001;127:207–13. 10.1017/S0950268801005957 11693497 PMC2869739

[2] Chiu CH, Wu TL, Su LH, Chu C, Chia JH, Kuo AJ, The emergence in Taiwan of fluoroquinolone resistance in *Salmonella enterica* serotype Choleraesuis. N Engl J Med. 2002;346:413–9. 10.1056/NEJMoa012261 11832529

[3] Farmer JJ III. *Enterobacteriaceae*: introduction and identification. In: Murray PR, Baron EJ, Pfaller MA, Tenover FC, Yolken RH, editors. Manual of clinical microbiology. 6th ed. Washington: American Society for Microbiology; 1995. p. 438–49.

[4] National Committee for Clinical Laboratory Standards. Performance standards for antimicrobial disk susceptibility tests for bacteria that grow aerobically. Approved standard M2-A7. 7th ed. Villanova (PA): The Committee; 2000.

[5] Centers for Disease Control and Prevention. *Salmonella* surveillance: annual summary, 2000. Atlanta: U.S. Department of Health and Human Services, Public Health Service; 2000.

[6] Chen YH, Peng CF, Tsai JJ, Hwang KP, Lu PL, Cheng HH, Epidemiological study of human salmonellosis during 1991–1996 in southern Taiwan. Kaohsiung J Med Sci. 1999;15:127–36. 10224836

[7] Chiu CH, Lin TY, Ou JT. Predictors for extraintestinal infections of non-typhoidal *Salmonella* in patients without AIDS. Int J Clin Pract. 1999;53:161–4. 10665125

[8] Chiu CH, Su LH, Chu C, Chia JH, Wu TL, Lin TY, Isolation of *Salmonella enterica* serotype choleraesuis resistant to ceftriaxone and ciprofloxacin. Lancet. 2004;363:1285–6. 10.1016/S0140-6736(04)16003-0 15094275

[9] Hsueh PR, Teng LJ, Tseng SP, Chang CF, Wan JH, Yan JJ, Ciprofloxacin-resistant *Salmonella enterica* Typhimurium and Choleraesuis from pigs to humans, Taiwan. Emerg Infect Dis. 2004;10:60–8. 15078598 10.3201/eid1001.030171 PMC3322755

[10] Chu C, Chiu CH, Wu WY, Chu CH, Liu TP, Ou JT. Large drug resistance virulence plasmids of clinical isolate of *Salmonella enterica* serovar Choleraesuis. Antimicrob Agents Chemother. 2001;45:2299–303. 10.1128/AAC.45.8.2299-2303.2001 11451688 PMC90645

